# Long-Term Breastfeeding: Protective Effects Against Triple-Negative Breast Cancer and the Role of the Breast Microbiota

**DOI:** 10.3390/pathogens14090946

**Published:** 2025-09-18

**Authors:** Julia Sierra-Roca, Joan Climent

**Affiliations:** 1 Department of Obstetrics and Gynecology, La Ribera Salud, 46015 Sueca, Spain; m.j.sierraroca@hotmail.com; 2 ECMOR Chair@CEU-UCH, Universidad Cardenal Herrera-CEU, CEU Universities, 46115 Alfara del Patriarca, Spain; 3 PASAPTA Department, Veterinary School, Universidad Cardenal Herrera-CEU, CEU Universities, 46115 Alfara del Patriarca, Spain

**Keywords:** breastfeeding, triple-negative breast cancer, TNBC, microbiota, breast cancer prevention, dysbiosis, lactation, probiotics, tumor microenvironment

## Abstract

Long-term breastfeeding is a modifiable, protective factor against breast cancer, particularly triple-negative breast cancer (TNBC), a highly aggressive subtype with limited therapeutic options. Recent findings suggest that the protective effects of breastfeeding are mediated not only through hormonal regulation and epithelial differentiation but also via the modulation of the breast microbiota. This review examines epidemiological data on the association between breastfeeding duration and TNBC risk reduction, highlights the composition and functions of breast microbiota in health and disease, and discusses how dysbiosis may contribute to carcinogenesis. By examining the microbiome’s role in maintaining breast health, we emphasize how breastfeeding contributes to a natural defense system against TNBC, offering a unique perspective on the intersection of maternal health, microbiology, and cancer prevention. Breastfeeding not only provides maternal and infant benefits but also emerges as a biological strategy that promotes cancer resistance through its influence on the breast microbiota. Integrating epidemiological, microbiological, and mechanistic evidence suggests that future research deliberately combining these domains will be essential to clarify causal pathways and translate findings into prevention and intervention strategies against TNBC.

## 1. Introduction

Breastfeeding is a natural and highly beneficial process that supports the health and well-being of both mothers and their infants. For infants, breastfeeding provides optimal nutrition, enhances immune development, and fosters the establishment of a healthy gut microbiome, which has long-term implications for immune and metabolic health [1,2].

For mothers, lactation accelerates postpartum recovery, including faster uterine involution and reduced postpartum bleeding, and helps support return to pre-pregnancy weight and emotional well-being. Long-term breastfeeding is associated with reduced risks of ovarian and endometrial cancers and has been linked to a lower incidence of type 2 diabetes and cardiovascular disease [3,4,5].

Notably, a growing body of evidence supports a protective role for breastfeeding against breast cancer, especially the aggressive triple-negative subtype (TNBC), which lacks expression of estrogen receptor (ER), progesterone receptor (PR), and human epidermal growth factor receptor 2 (HER2). As a clinically challenging subtype, TNBC presents limited targeted treatment options and a poorer prognosis than other breast cancer subtypes [6,7].

TNBC is more frequently diagnosed in younger, premenopausal women and is particularly prevalent among women of African descent [8,9,10]. Several epidemiological studies have found that shorter durations of breastfeeding, or the absence of breastfeeding altogether, are associated with an elevated risk of developing TNBC. These findings underscore breastfeeding as a powerful and modifiable factor in breast cancer prevention, with important implications for reducing racial disparities in TNBC incidence.

In parallel, there is growing interest in the human breast microbiota, an ecosystem of bacteria residing in the mammary glands and milk, and its possible roles in tissue homeostasis and disease development [10]. Once thought to be sterile, the breast is now known to harbor distinct microbial communities, with evidence suggesting that lactation, delivery method, and maternal gut microbiota influence its composition [10,11]. Disruption of this microbial balance, or dysbiosis, has been linked to inflammation, immune dysfunction, and carcinogenesis [12]. Furthermore, bacterial metabolites and immune signaling pathways mediated by the breast microbiota may influence the development of TNBC and other breast cancer subtypes [13].

Emerging research has identified distinct bacterial profiles in both milk and mammary tissue, with direct breastfeeding associated with beneficial taxa like *Lactobacillus*, *Bifidobacterium*, and *Streptococcus* [14,15]. In contrast, milk pumping or non-lactation is linked with a higher prevalence of potentially pathogenic *Enterobacteriaceae* and *Pseudomonas* species [10,15,16]. These microbial differences correlate with immune modulation, inflammatory status, and even histological changes in the mammary gland, factors intimately linked to carcinogenesis [17,18].

Additionally, studies in murine models have shown that abrupt involution, mimicking early weaning or non-breastfeeding, induces local inflammation, signal transducer and activator of transcription 3 (STAT3) activation, ductal hyperplasia, and expansion of luminal progenitor cells, which are thought to be the origin of basal-like and triple-negative tumors [18,19]. These findings support a paradigm where breastfeeding not only reduces risk through hormonal and structural pathways but also establishes a resilient microbial ecosystem in the breast that fosters immune tolerance and epithelial stability [18,19].

This review explores the intersection between long-term breastfeeding, breast microbiota, and TNBC risk. We synthesize findings from epidemiological studies, murine models, microbiome analyses, and immune profiling to propose an integrative framework whereby breastfeeding shapes a favorable immune and microbial microenvironment that reduces TNBC risk. By articulating the biological and public health relevance of this axis, we aim to highlight novel pathways for cancer prevention and identify opportunities for clinical and translational research.

## 2. Epidemiological Evidence Linking Breastfeeding and TNBC Risk

A substantial body of epidemiological evidence supports the protective role of long-term breastfeeding in reducing the risk of TNBC [4,8,20]. TNBC is a particularly aggressive subtype with limited therapeutic options, contributing to poor prognosis [5]. A comprehensive meta-analysis by Kumar et al., including 33 studies, demonstrated that ever having breastfed was associated with a significant ~20% reduction in TNBC risk (OR = 0.80, 95% CI: 0.66–0.98) [21]. Similarly, the systematic review and meta-analysis by Mao et al. [7], covering over 75 studies, confirmed that breastfeeding was consistently associated with decreased risk across molecular subtypes, with a notably strong inverse relationship for TNBC. These analyses underscore breastfeeding as a robust protective factor, particularly for this basal-like and therapeutically challenging subtype.

The specificity of this association becomes more apparent when comparing reproductive risk profiles across subtypes. While parity is generally protective of luminal subtypes, several studies have shown that multiparity without breastfeeding increases the risk of TNBC, potentially due to abrupt post-pregnancy involution and inflammation [22,23,24]. This suggests that breastfeeding is not only beneficial on its own but also mitigates the risks otherwise conferred by multiple pregnancies.

In a population-based study, Chehayeb et al. [6] quantified the public health impact of breastfeeding on TNBC incidence. Their analysis estimated that 12% of TNBC cases in White women and 15% in Black women in the United States could be attributed to breastfeeding for less than six months. Among women aged 20–44, this proportion rose to 18%, illustrating the vulnerability of younger women [25]. These findings align with those of the Carolina Breast Cancer Study and others, which have consistently demonstrated racial disparities in breastfeeding practices that mirror TNBC incidence patterns [25,26]. These results are consistent with earlier findings by Millikan et al., who reported a combined population attributable fraction (PAF) of 53% for TNBC due to never breastfeeding and an elevated waist-to-hip ratio, reflecting central obesity and visceral adiposity, which promote chronic inflammation, insulin resistance, and increased estrogen production, all of which contribute to breast cancer risk [27]. Although older studies did not isolate TNBC from other subtypes, their conclusions support the growing consensus that breastfeeding is a powerful protective factor, particularly against aggressive, hormone-independent breast cancers [6,24].

Importantly, breastfeeding appears to be protective even in high-risk groups. For instance, Islami et al. reported that among breast cancer susceptibility gene 1 (BRCA1) mutation carriers, who are predisposed to triple-negative and basal-like tumors, long-term breastfeeding was associated with a reduced risk of breast cancer overall, with the most substantial reductions observed in basal-like subtypes [28]. Supporting this, Kotsopoulos et al. found that breastfeeding for more than one year reduced breast cancer risk by 32% in BRCA1 mutation carriers [29]. Mechanistically, animal and human studies suggest that prolonged breastfeeding promotes the gradual involution of the mammary gland, reducing inflammation and limiting the expansion of TNBCs, the presumed cell of origin for basal-like tumors in BRCA1 carriers [19,30]. In contrast, abrupt involution, typical of short or absent lactation, induces STAT3 activation, collagen remodeling, immune infiltration, and Notch signaling pathway activation, hallmarks of a pro-tumorigenic environment associated with TNBC and basal-like cancers [19,31]. These findings provide a compelling biological framework for the observed epidemiological associations.

Additional studies have highlighted the duration-dependent nature of this protective effect [32,33,34]. The Women’s Circle of Health Study and several pooled case–control analyses have shown that the longer the cumulative duration of breastfeeding, the greater the reduction in TNBC risk, though a clear threshold or dose–response relationship remains to be conclusively defined due to heterogeneity across studies [22,24]. Meta-analyses indicate a ~4.3% decreased breast cancer risk per 12 additional months of breastfeeding [35], and in pooled analyses, African-American women who breastfed ≥6 months had up to an 82% lower risk of TNBC (OR = 0.18) [24].

In summary, breastfeeding is a modifiable, cost-effective behavior that significantly reduces TNBC risk. Its protective effects are consistent across racial and genetic backgrounds and particularly important for mitigating the impact of multiparity and promoting equitable cancer prevention. These findings support the inclusion of breastfeeding history in breast cancer risk models and call for public health strategies targeting disparities in breastfeeding access and education.

## 3. Breast Microbiota: Composition and Function in Health

The human breast is home to a diverse and dynamic microbial community that plays a critical role in local immune regulation, epithelial homeostasis, and potentially cancer prevention [10,18,36]. Once considered sterile, breast tissue and milk are now recognized as distinct microbial niches influenced by host factors such as lactation, mode of delivery, maternal gut microbiota, infant sex, and breastfeeding practices [16,18,37]. The origin of the breast microbiota is hypothesized to involve two primary mechanisms: entero-mammary translocation, where bacteria from the maternal gut migrate to the mammary gland via dendritic cells and macrophages, even before breastfeeding begins [38,39]; and retrograde inoculation, where infant oral microbes are transferred into the nipple ducts during breastfeeding [11,16,40]. Supporting this, microbial DNA has been detected in colostrum before first feeding, indicating a prenatal microbial transfer [41]. In addition, the similarity between infant oral and milk microbiota, and the distinct patterns observed in milk collected via pumping vs. direct breastfeeding, supports the retrograde inoculation hypothesis [11,16]. The structure of the breast microbiota may thus reflect both endogenous sources and external inoculation, shaping the microbial and immune landscape of the mammary gland [10,40,41].

### 3.1. Composition of Breast Microbiota

Culture-independent sequencing technologies have consistently revealed a core microbiome in healthy breast tissue and milk, composed predominantly of *Staphylococcus*, *Streptococcus*, *Corynebacterium*, *Propionibacterium*, *Lactobacillus*, and *Bifidobacterium* [10,18,41,42]. These taxa are not only commensal but may also contribute to epithelial integrity, immune tolerance, and nutrient metabolism, supporting both maternal breast health and infant development [14,15,42].

Breastfeeding status is a major determinant of microbial diversity and composition. Direct breastfeeding has been associated with a higher abundance of beneficial genera such as *Veillonella*, *Lactobacillus*, and *Bifidobacterium*, all of which support infant gut colonization and immune development [17,43,44]. In contrast, milk obtained via pumping shows increased presence of opportunistic or potentially pathogenic taxa, including *Enterobacteriaceae*, *Pseudomonas*, and *Stenotrophomonas*, as well as reduced microbial richness and altered community structure [17,43,45]. These compositional differences likely result from retrograde inoculation during nursing and environmental contamination during pumping. In addition to the feeding method, several maternal and perinatal factors, such as mode of delivery, maternal antibiotic exposure, gestational age at birth, maternal BMI, lactation stage, and infant sex, contribute to interindividual variation in breast milk microbiota, though their effects tend to be less pronounced or context-specific [16,40,41]. Structural equation modeling of the CHILD cohort data confirmed that mode of breastfeeding was one of the strongest independent predictors of milk microbial composition, reinforcing the key role of nursing behaviors in shaping this ecosystem [16]. Multiple studies have investigated microbial communities in breast tissue, human milk, and the gut, each providing evidence of associations with breast cancer risk and progression. To provide an overview of this body of work, we summarize the main findings, methodologies, and subtype-specific associations in Supplementary Table S1.

Recent studies suggest these microbial differences may have biological consequences. The “Anna Karenina principle” (AKP) has been proposed to explain increased microbial heterogeneity in breast disease, showing that breast cancer is associated with less variation in rare taxa (anti-AKP effect), contrasting the greater stochasticity seen in mastitis [46]. Furthermore, a 2025 meta-analysis revealed that microbial alpha diversity is significantly decreased in breast tumor tissues, especially in TNBC, while taxa such as *Fusobacteriota*, *Peptoniphilus*, and *Atopobium* are enriched in tumors compared to adjacent healthy tissue [45]. The differential presence of specific bacteria, including *Bacillus thermoamylovorans*, has even been shown to enhance breast cancer metastasis in murine models, highlighting the possibility that certain bacterial species actively contribute to tumor progression through direct modulation of cancer cell metabolism and immune signaling [47].

**Figure 1:**
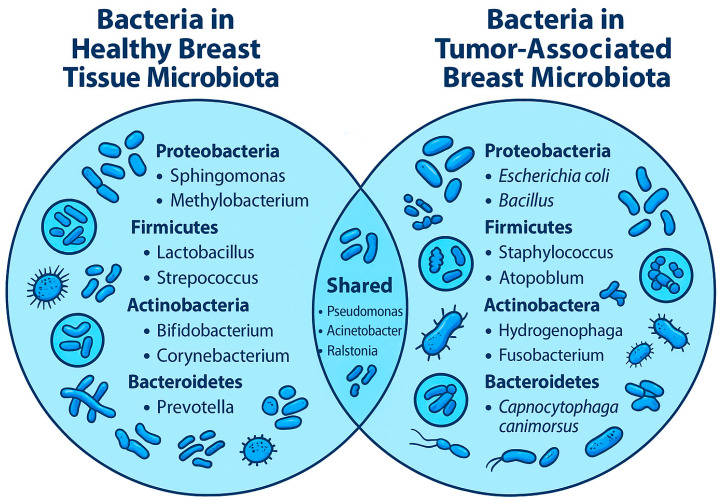
A Venn diagram illustrates the intersection of bacterial taxa between healthy and tumor-associated breast tissue microbiota. This diagram compares the breast microbiota identified in healthy tissue and tumor tissue from breast cancer patients, with the overlapping area representing bacterial genera and families found in both. Healthy tissue microbiota includes genera such as *Lactobacillus*, *Streptococcus*, *Bifidobacterium*, *Methylobacterium*, *Sphingomonas*, *Prevotella*, and *Propionibacterium*, often associated with immune regulation, epithelial integrity, and low inflammation. Tumor tissue microbiota features genera like *Staphylococcus*, *Escherichia*, *Bacillus*, *Ralstonia*, *Atopobium*, and *Paenibacillus*, some of which are linked to inflammation, immune evasion, or cancer progression. Shared taxa include *Pseudomonas*, *Corynebacterium*, *Propionibacterium*, *Ralstonia*, *Streptococcus*, and *Lactobacillus*, though their abundance and potential functional roles differ markedly between tissue types. The figure emphasizes how microbial dysbiosis, rather than the presence or absence of specific taxa alone, may play a critical role in breast carcinogenesis. Illustrative bacteria are included for visual reference and do not represent actual proportions. This representation summarizes findings from multiple studies examining the microbiota in both healthy breast tissue and breast tumors.

Current deep sequencing and microbial profiling studies have confirmed the dominance of *Proteobacteria* and *Firmicutes* in healthy breast tissue, with families such as *Acetobacteraceae*, *Lactobacillaceae*, and *Xanthomonadaceae* showing high abundance in cancer-free individuals [42,48,49]. Specifically, *Acetobacter aceti*, *Liquorilactobacillus paracasei* (a reclassified species from *Lactobacillus*), and members of *Xanthomonas* have been associated with younger age, parity, and favorable immune and metabolic gene expression in adjacent transcriptomic profiles [10,42,48,49]. In contrast, breast cancer tissues, including those from TNBC, demonstrate consistent enrichment in *Ralstonia* and *Escherichia coli*, species linked to pro-inflammatory signaling, oxidative stress, and DNA damage, all of which contribute to tumor progression and genomic instability [10,11,12,13,42,50]. These compositional differences between healthy and malignant breast tissue (Figure 1) not only reinforce the concept of microbial dysbiosis in carcinogenesis but also suggest that specific taxa may modulate host immune responses and influence cancer risk at the molecular level [12,47,50,51].

In non-lactating women, particularly those who are postmenopausal or nulliparous, breast tissue exhibits distinct microbial profiles characterized by reduced microbial diversity and increased prevalence of pro-inflammatory and potentially pathogenic taxa such as *Escherichia coli*, *Staphylococcus aureus*, and *Bacillus* spp. [10,18,52,53]. These bacteria have been shown to induce DNA damage and promote inflammatory responses. For example, *E. coli* and *Staphylococcus epidermidis* isolated from breast tumors can induce double-strand DNA breaks, potentially contributing to genomic instability [10,50,52]. Moreover, *Streptococcus pyogenes* and other β-glucuronidase-producing bacteria may influence local estrogen metabolism, facilitate estrogen reactivation, and promote oncogenic signaling, especially in hormone-sensitive tissues [54,55]. These dysbiotic patterns are more frequently observed in cancerous breast tissue compared to adjacent normal tissue, suggesting a parallel shift in microbial composition with disease progression [10,53,56].

### 3.2. Functions of the Breast Microbiota

Beyond compositional characteristics, the functional roles of breast microbiota are critical for maintaining mammary gland homeostasis and may contribute to protection against triple-negative breast cancer (TNBC). Beneficial commensals, particularly *Lactobacillus* and *Bifidobacterium*, contribute to epithelial barrier integrity, competitive exclusion of pathogens, and local immune regulation through the secretion of immunomodulatory compounds [18,48,57,58,59]. Among the most biologically relevant of these are short-chain fatty acids (SCFAs), including butyrate, acetate, and propionate, which can influence both local and systemic immunity [60,61]. SCFAs act as histone deacetylase (HDAC) inhibitors, modulating gene expression through epigenetic mechanisms. In breast cancer models, sodium butyrate has been shown to induce cell cycle arrest and apoptosis, reduce expression of oncogenes like cellular myelocytomatosis oncogene (c-MYC), and increase tumor suppressor genes such as *p21* [60,61]. These effects are particularly important in TNBC, a with limited treatment options; the ability of bacterial metabolites to suppress cell proliferation and promote tumor cell differentiation offers a promising, non-hormonal avenue of prevention [62,63,64]. In addition, commensal bacteria contribute to immune homeostasis by supporting the development of regulatory T cells, suppressing pro-inflammatory cytokines, and enhancing antigen presentation and epithelial tolerance [18,47]. Experimental studies suggest that alterations in breast microbiota composition may either promote or suppress immune-mediated tumor surveillance, depending on the balance between immunoregulatory and pro-inflammatory microbial metabolites and taxa [47,48,58]. Collectively, these functions highlight the biological relevance of the breast microbiota in maintaining epithelial integrity, modulating immune signaling, and regulating gene expressions, all of which may influence TNBC development and progression, independently of hormone-driven pathways.

Recent integrative analyses have revealed that microbial abundance in normal breast tissue correlates with host gene expression signatures, particularly immune and metabolic pathways. In a large-scale profiling of 403 healthy breast tissue samples and adjacent normal tissues from breast cancer patients, *Lactobacillus vini*, *Lactobacillus paracasei*, *Acetobacter aceti*, and *Xanthomonas* species were found to associate with upregulation of genes involved in immune signaling, fatty acid metabolism, and epithelial homeostasis. Genes involved in immune signaling (e.g., *IL2*, *IFNG*, *CXCL10*), fatty acid metabolism (e.g., *FASN*, *ACACA*), and epithelial homeostasis (e.g., *CDH1*, *OCLN*) were among those correlated with microbial abundance [48,52,53]. Among the taxa enriched in breast tissue, *Ralstonia* has been consistently associated with a dysbiotic profile. Its abundance correlates with transcriptomic signatures of carbohydrate metabolism, including glycolytic and pentose phosphate pathway genes, and with downregulation of interferon- and cytokine-mediated signaling [48,49]. These alterations suggest that *Ralstonia* may contribute to immune evasion in the mammary microenvironment. In contrast, protective genera such as *Lactobacillus* and *Bifidobacterium* are associated with enhanced epithelial barrier function, stimulation of regulatory T cells, and the induction of anti-inflammatory cytokines, including interleukin-10 (IL-10) and transforming growth factor beta (TGF-β). Together, these data highlight that specific microbial shifts can differentially modulate metabolic and immune pathways relevant to TNBC risk. The transcriptomic patterns associated with high Ralstonia abundance in breast tissue reveal changes in gene expression that are indicative of immune modulation and potential immune evasion. Specifically, German et al. (2023), found that microbial levels correlate with alterations in immune-related pathways, including IL17 signaling, T cell receptor signaling, and inflammatory responses [48]. The observed gene expression changes suggest that elevated Ralstonia levels might contribute to an immunosuppressive environment, impairing the tissue’s ability to detect and eliminate early tumor cells. This immune evasion could facilitate the progression from normal tissue to tumor by allowing abnormal cells to evade immune destruction, thereby increasing breast cancer risk. These findings support the concept that microbial dysbiosis in the mammary gland not only reflects disease but may actively contribute to the transcriptomic reprogramming associated with tumor microenvironments [65,66]. Several bacterial taxa are found in both healthy and tumor-associated breast tissues, but with differing abundances and potential functions [52,53,66]. This taxonomic context complements recent integrative analyses demonstrating how microbial presence correlates with host gene expression signatures related to immune modulation, metabolic reprogramming, and tumorigenic pathways [66].

**Figure 2:**
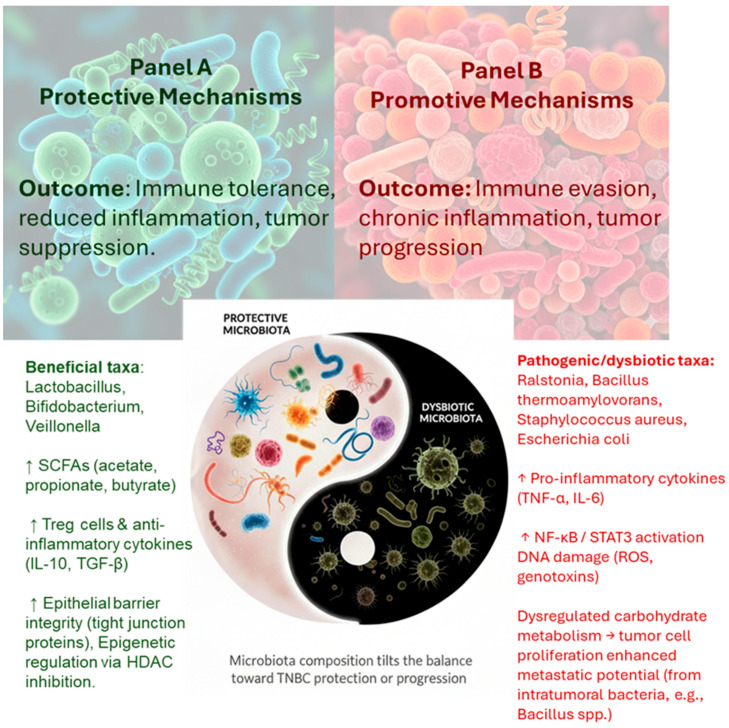
Protective and promotive mechanisms of the breast microbiota in TNBC. Beneficial taxa such as *Lactobacillus* and *Bifidobacterium* produce short-chain fatty acids (SCFAs), enhance regulatory T cell activity, increase IL-10 and TGF-β secretion, and maintain epithelial barrier integrity, thereby contributing to immune tolerance and tumor suppression. In contrast, dysbiotic taxa including *Ralstonia*, *Staphylococcus aureus*, and intratumoral *Bacillus* species promote pro-inflammatory cytokine release (TNF-α, IL-6), activation of NF-κB and STAT3 signaling, DNA damage, and metabolic reprogramming, ultimately favoring tumor growth, immune evasion, and metastasis. The balance between protective and pathogenic microbes may critically influence TNBC occurrence and progression [18,47,52].

Microbial signals significantly shape immune responses in the mammary gland. Exposure to commensal bacteria such as *Lactobacillus* spp. can induce anti-inflammatory cytokines like IL-10 and TGF-β, fostering an immune-tolerant microenvironment that limits chronic inflammation and supports epithelial homeostasis (Figure 2) [10,12,18,19]. In contrast, microbial dysbiosis in breast tissue is associated with increased levels of pro-inflammatory cytokines such as IL-6, tumor necrosis factor alpha (TNF-α), and IL-1β. These cytokines can activate oncogenic signaling pathways, including nuclear factor kappa-light-chain-enhancer of activated B cells (NF-κB) and STAT3, contributing to a pro-tumorigenic microenvironment and cancer progression [18,19,54,67]. Notably, in vivo studies in mice demonstrate that the administration of beneficial bacteria (e.g., *Lactobacillus helveticus* or *Lactobacillus acidophilus*) modulates the mammary immune profile by increasing IL-10 and reducing IL-6, thereby suppressing tumor growth and inflammation [68,69].

The tumor microenvironment is increasingly recognized as being shaped by the presence and activity of intratumoral bacteria. Recent evidence from Gerbec et al. demonstrated that *Bacillus thermoamylovorans*, a species isolated from metastatic TNBC tumors, significantly enhanced metastatic burden in preclinical TNBC models [47]. Tumor cells co-cultured with *B. thermoamylovorans* exhibited substantial alterations in metabolic pathways, including enriched amino acid and nucleotide metabolism, both of which are associated with metastatic progression [47]. These effects were specific to *B. thermoamylovorans*, as co-culture with non-metastatic isolates such as *B. subterraneus* did not replicate this phenotype. Furthermore, genomic analysis revealed that *B. thermoamylovorans* harbored unique functional gene signatures associated with disease progression and mortality in patient samples [47]. These findings establish a functional link between intratumoral microbiota composition and breast cancer metastasis, underscoring the importance of microbial contributions to tumor biology.

Overall, the breast microbiota represents a key regulator of tissue homeostasis and immune tone in the mammary gland. Its composition and activity are shaped by lactation, which serves not only to feed the infant but also to reinforce a beneficial microbial-immune axis in the mother. Disruption of this axis, through abrupt weaning, formula feeding, or antibiotic exposure, may contribute to the development of a pro-tumorigenic environment in the breast. Understanding these mechanisms may inform future interventions, including microbiota-based therapies, precision probiotic supplementation, or microbial biomarkers for early cancer detection.

## 4. Long-Term Breastfeeding and Breast Cancer Risk Reduction

A growing body of epidemiological evidence supports the association between long-term breastfeeding and a reduced risk of breast cancer, particularly for the TNBC subtype (Table 1) [4,6,7,19,21,26,28,34,70]. This protective effect appears to be multifactorial, encompassing hormonal regulation, immune modulation, metabolic reprogramming, and possibly epigenetic remodeling. Studies have shown that breastfeeding for more than 12 months can lower the risk of TNBC by up to 42% compared to parous women who do not breastfeed [71]. Importantly, shorter durations of lactation (e.g., breastfeeding for <6 months) have been linked to increased TNBC risk, with pooled odds ratios ranging from 1.4 to 2.0 depending on parity and race [26]. The proposed mechanisms include decreased exposure to cyclic estrogen and progesterone, enhanced differentiation of mammary epithelial cells, modulation of inflammation during gradual gland involution, and a persistent imprint on the epigenetic landscape of the mammary gland [26,34].

Building upon the epidemiological findings in Section 2, the Carolina Breast Cancer Study (CBCS) and other large-scale cohorts, including the AMBER Consortium, Black Women’s Health Study (BWHS), and Women’s Circle of Health Study (WCHS), have consistently shown that breastfeeding substantially mitigates the increased TNBC risk associated with parity in Black women. For example, parous women who had not breastfed had a 68% higher risk of TNBC, which was nullified if they breastfed [24]. These findings support a significant role of breastfeeding in reducing racial disparities in TNBC incidence.

Moreover, lack of breastfeeding leads to abrupt involution, characterized by a pro-inflammatory environment, enhanced collagen remodeling, and dysregulated tissue architecture. Epidemiological studies support these mechanistic findings, showing that women who did not breastfeed or underwent abrupt weaning are at increased risk of basal-like/TNBC [19]. The biological mechanisms underlying this association are described in Section 3.1. These changes were associated with ductal hyperplasia, squamous metaplasia, and activation of Notch signaling, recapitulating features found in basal-like and TNBC tumors [19].

In parallel, exosomal microRNAs (miRNAs) present in breast milk have been implicated in the epigenetic regulation of mammary epithelial cells. Specifically, milk-derived miR-29 and miR-148a target DNA methyltransferase DNMT3a/b, and DNMT1, respectively, thereby modulating DNA methylation patterns relevant to lactation and cellular differentiation [72]. Decreased exposure to these miRNAs due to early weaning or formula feeding may impair the establishment of stable epigenetic marks in the mammary epithelium, potentially increasing susceptibility to malignancy [72,73].

The hormonal and metabolic shifts during lactation also have protective effects. Prolonged lactation reduces circulating levels of estrogen and prolactin, hormones known to stimulate breast tissue proliferation [74]. Moreover, breastfeeding promotes metabolic adaptations including enhanced insulin sensitivity and lipid utilization, which collectively reduce the risk of obesity, a known risk factor for postmenopausal breast cancer [75].

In summary, long-term breastfeeding is a powerful, modifiable protective factor against breast cancer and particularly TNBC. Its effects appear to span biological pathways including epithelial differentiation, immune regulation, hormone suppression, and epigenetic remodeling. Addressing disparities in breastfeeding rates, through education, policy changes, and community support, could reduce TNBC incidence and improve maternal health outcomes across populations.

## 5. Integrative Mechanisms Linking Breastfeeding, Microbiota, and TNBC Protection

Building upon the compositional insights of the breast microbiota, growing evidence suggests that breastfeeding shapes a dynamic immuno-metabolic environment that may protect against TNBC through several interconnected biological pathways. These include hormone modulation, microbial-mediated epigenetic changes, and long-term immune imprinting [6,76].

A novel aspect gaining attention is the intergenerational influence of breastfeeding-mediated microbial transfer. Colonization of the infant gut with maternally derived bacteria such as *Bifidobacterium breve* and *Lactobacillus rhamnosus* may promote early immune education, reduce systemic inflammation, and even influence long-term estrogen metabolism. These lifelong effects underscore breastfeeding not only as a maternal protective behavior but also as a foundational mechanism in shaping immune resilience in the next generation [16,20,77].

**Figure 3:**
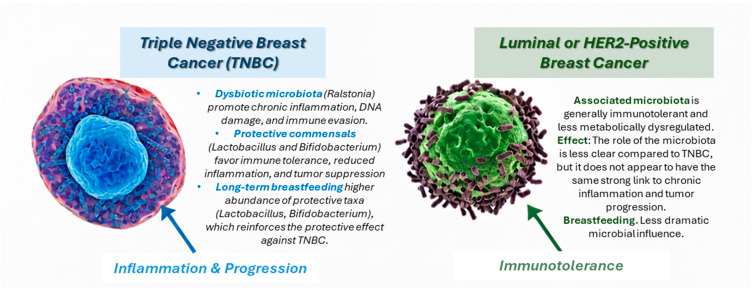
Microbiota’s Influence on Breast Cancer Subtypes. This figure illustrates the contrasting roles of the tumor microbiota in TNBC versus Luminal or HER2-positive breast cancers. Left Side (TNBC): Dysbiotic microbiota, characterized by a predominance of pathogenic species, drives an inflammatory environment. This leads to tumor progression, enhanced metastatic potential, and a less favorable prognosis. This side is associated with factors like the absence of breastfeeding, which influences the microbial balance in a way that favors these harmful organisms. Right Side (Luminal or HER2-positive Breast Cancer): This tumor type is associated with a more immunotolerant microbiota. This microbial community does not promote the same level of chronic inflammation or metabolic dysregulation as seen in TNBC, leading to a different tumor progression pathway. The figure’s central point of comparison highlights how specific microbial compositions can tilt the balance toward either protection or progression, emphasizing the distinct mechanisms at play in different breast cancer subtypes.

Recent studies also highlight how microbial signatures differ across breast cancer subtypes (Figure 3). Epidemiological studies further support these mechanistic observations, showing that long-term breastfeeding reduces the prevalence of dysbiotic taxa such as *Ralstonia*, while favoring enrichment of protective commensals like *Lactobacillus* and *Bifidobacterium* [47,48,49]. This microbial balance appears to influence both inflammatory responses and epithelial homeostasis, reinforcing the protective effect of breastfeeding against TNBC. To avoid redundancy, the mechanistic details of microbial functions are discussed in Section 3.2, and here we focus on the epidemiological associations [78,79,80]. By contrast, the microbiota associated with luminal or HER2-positive tumors tends to be more immunotolerant and less metabolically dysregulated [54,66,81].

The potential of breastfeeding to shift the breast microbiota away from tumor-promoting profiles, either directly through microbial exposure or indirectly through structural, immune, and hormonal changes, offers a compelling integrative model of TNBC prevention [9,22,33]. In this framework, breastfeeding acts not only as a physiological reset after pregnancy but also as a microbial and immunological calibrator that helps maintain epithelial stability and suppress carcinogenic transitions [16,34].

**Figure 4:**
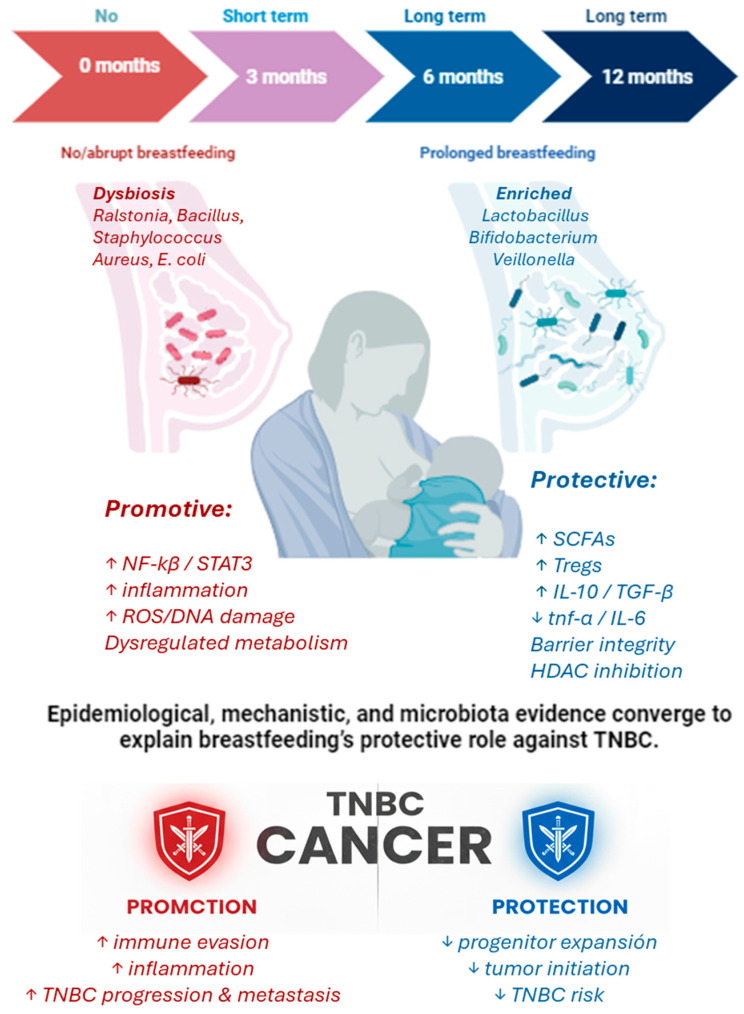
Integrative model linking breastfeeding, microbiota, and TNBC protection. This conceptual framework illustrates how breastfeeding practices shape the breast and milk microbiota, influencing host immune, inflammatory, and metabolic pathways that ultimately affect TNBC risk. Prolonged breastfeeding promotes the enrichment of beneficial taxa such as *Lactobacillus* and *Bifidobacterium*, which increase short-chain fatty acid (SCFA) production and contribute to a stable, diverse microbial community. These microbes support immune tolerance by enhancing regulatory T cells and anti-inflammatory cytokines (IL-10, TGF-β), reducing pro-inflammatory mediators (TNF-α, IL-6), maintaining epithelial barrier integrity, and exerting epigenetic control through HDAC inhibition. Together, these mechanisms reduce luminal progenitor expansion, inflammation, and DNA damage, thereby lowering TNBC risk. Conversely, absence of or abrupt cessation of breastfeeding is associated with dysbiosis, characterized by the enrichment of taxa such as *Ralstonia*, *Bacillus*, *Staphylococcus*, and *Escherichia coli*. These microbial profiles favor chronic inflammation, NF-κB/STAT3 activation, DNA damage, immune evasion, and metabolic dysregulation, leading to enhanced tumor progression and metastasis. The model highlights how the balance between prolonged breastfeeding with a healthy microbiota and short or absent breastfeeding with dysbiosis can tip the trajectory toward either protection or promotion of TNBC.

Finally, understanding how breast microbiota evolves throughout pregnancy, lactation, and involution remains a key research priority. Longitudinal studies tracking these microbial and immuno-hormonal dynamics in high-risk women could elucidate critical windows for intervention. Identifying microbial markers that predict TNBC risk, or that reflect early carcinogenic changes, may also inform future diagnostic and preventive strategies. Figure 4 presents an integrative model illustrating how breastfeeding influences the microbiota, thereby shaping immune and metabolic pathways that determine TNBC protection or progression. The integrative mechanisms illustrated in Figure 2, Figure 3 and Figure 4 are supported by epidemiological and microbiota evidence summarized in Supplementary Table S1.

## 6. Therapeutic and Preventive Implications

Advancements in our understanding of how breastfeeding and breast microbiota influence the risk of TNBC are opening promising avenues for innovative prevention and treatment strategies. Given the lack of hormone or HER2 targets in TNBC, these microbiota-centered approaches may serve as crucial adjuncts to conventional treatments.

One area of growing interest is the targeted modulation of the breast and gut microbiome. Preclinical research has demonstrated that enriching beneficial microbial taxa, such as *Lactobacillus*, *Bifidobacterium*, *Alistipes*, and *Ruminococcus*, can stimulate anti-tumor immunity, enhance IL-12 production, and activate CD8^+^ T cells, thereby reshaping the tumor immune microenvironment toward a more immunostimulatory state [69,82,83,84]. Conversely, reducing pro-inflammatory bacteria, such as Ralstonia or Escherichia coli, may alleviate immunosuppressive signaling cascades. Intratumoral microbes, such as *Bacillus thermoamylovorans*, identified in metastatic TNBC, further underscore the potential for microbiome-directed interventions to hinder metastasis [47].

Diet and probiotics have also emerged as powerful tools in this context. Probiotic strains including *L. plantarum*, *L. casei*, *L. acidophilus*, and *L. rhamnosus* have demonstrated tumor-suppressive activity in animal models by promoting macrophage and T cell activation and reducing oxidative damage and chronic inflammation [69,82,83,84]. Ongoing clinical trials (e.g., NCT03290651, NCT04362826) are investigating the effects of oral probiotic supplementation on gut and breast microbiota composition, immune responses, and cancer-related outcomes [13,85,86]. Moreover, diet-based interventions rich in polyphenols, prebiotic fibers, and fermented foods can enhance the abundance of SCFA-producing microbes and microbial diversity [80,87,88]. Synbiotics, combinations of probiotics and prebiotics, are being evaluated for their potential synergistic effects, although robust human data are still emerging.

Beyond therapeutic benefit, breastfeeding itself plays a critical preventive role through microbial transmission. Human milk is a key vehicle for transferring beneficial bacteria such as *Bifidobacterium breve* and *Lactobacillus rhamnosus*, which promote early immune tolerance and may influence systemic inflammation and estrogen metabolism in offspring. This intergenerational transmission underscores breastfeeding not only as a maternal protective factor but also as a foundation for long-term health in the next generation [59].

Microbiome profiling may also offer predictive and prognostic insights in oncology. Characterizing gut and breast microbiota before and after treatment could help stratify patients by likely response to chemotherapy, endocrine therapy, or immunotherapy [88,89]. Experimental models suggest that probiotics like *Lactobacillus johnsonii* may enhance the efficacy of immune checkpoint inhibitors such as anti–PD-1 in breast cancer [90].

Looking ahead, next-generation tools such as bacteriotherapy (the administration of live beneficial bacteria) and microbiota-derived bioactive peptides, like colicins and defensins, are being investigated for their ability to directly modulate immune responses and suppress tumor growth in preclinical systems [68]. These innovative approaches hold promise for expanding the therapeutic arsenal in TNBC, particularly in patients who do not respond to standard regimens.

Research priorities moving forward include longitudinal studies to monitor shifts in the breast microbiota across pregnancy, lactation, and involution, especially in high-risk populations. There is also a need to develop precision synbiotics designed to increase SCFA-producing and anti-inflammatory taxa while suppressing β-glucuronidase–expressing microbes implicated in local estrogen reactivation. Finally, incorporating microbiota-supportive strategies into public health and breastfeeding promotion efforts could not only reduce TNBC disparities but also broadly improve maternal and child health outcomes. Together, these microbiota-centered strategies underscore the therapeutic potential of targeting the microbial-immune interface in breast cancer, particularly for TNBC, where conventional options remain limited. Harnessing the power of the microbiome, through breastfeeding, diet, and therapeutic modulation, may offer transformative opportunities for cancer prevention and patient care.

## 7. Future Perspectives and Directions

Although long-term breastfeeding has been consistently associated with reduced risk of triple-negative breast cancer (TNBC), many critical questions remain regarding the biological mechanisms and the role of microbiota in mediating this effect. While previous works have separately demonstrated that breastfeeding protects against TNBC, that breast tissue harbors a microbiome, and that lactation shapes microbial and immune environments, our review is, to our knowledge, the first to bring these strands together. We specifically propose that the protective effect of breastfeeding may be mediated, at least in part, through its influence on breast microbiota, thereby linking epidemiological observations to mechanistic evidence. This integrative perspective highlights a novel research frontier.

To advance the field, prospective longitudinal studies are urgently needed. Such studies should track breastfeeding duration, involution patterns (gradual vs. abrupt), and microbiota composition across diverse populations, ideally beginning during pregnancy and extending through weaning and postpartum. These designs will help clarify temporal dynamics and establish causal links.

The development of microbiota-based biomarkers represents another promising avenue. Microbial signatures derived from breast tissue, milk, or even stool samples could complement genetic and clinical risk factors for TNBC, enabling earlier detection or refined risk stratification, especially among BRCA1 carriers or women with strong family histories.

Parallel efforts should focus on microbiome-targeted interventions. Preclinical evidence suggests that probiotics, prebiotics, synbiotics, and dietary modulation can enrich beneficial taxa and reduce pro-inflammatory signaling. Translating these findings into clinical prevention strategies for at-risk women will require carefully designed, culturally sensitive, and ethically sound trials.

At the mechanistic level, multi-omics integration will be crucial to understanding how microbial metabolites, immune signaling, and host epigenetic regulation interact to modulate TNBC risk. Combining genomics, transcriptomics, epigenomics, metabolomics, and microbiota profiling will enable the identification of causal pathways and potential therapeutic targets.

Finally, public health initiatives remain central. Supporting breastfeeding through inclusive, equitable, and culturally sensitive policies is a natural and cost-effective intervention with intergenerational benefits. Embedding microbiome science into oncology, maternal care, and prevention programs could amplify these gains, particularly in populations disproportionately affected by TNBC.

Together, these future directions underscore the need for integrative, translational, and population-based research. Such approaches will move the field from descriptive associations to actionable strategies that combine maternal care, microbial science, and oncology for the prevention of TNBC.

## 8. Conclusions

This review highlights the multifactorial role of long-term breastfeeding in reducing the risk of TNBC. Beyond hormonal regulation and epithelial remodeling, breastfeeding actively shapes the breast microbiota, influencing local immunity, inflammation, and tissue homeostasis in ways that appear protective against carcinogenesis.

Breastfeeding should be recognized not only as maternal care but also as a biological strategy that promotes cancer resistance, microbial balance, and immune resilience. By linking epidemiological evidence, mechanistic insights, and microbiota research, this review underscores the novelty of integrating these perspectives and the promise they hold for TNBC prevention. Looking forward, future work that deliberately combines these three domains, breastfeeding behaviors, breast and milk microbiota composition, and molecular mechanisms of carcinogenesis, will be essential to establish causality and to translate these insights into clinical and public health strategies. Such integrative approaches may ultimately yield microbiota-informed biomarkers, prevention tools, and therapeutic targets that complement breastfeeding as a natural and cost-effective means of reducing TNBC risk.

## Figures and Tables

**Table 1:** Epidemiological evidence linking breastfeeding duration with breast cancer risk, with emphasis on triple-negative breast cancer (TNBC). This table summarizes major cohort, case–control, and pooled analyses evaluating the association between breastfeeding and breast cancer risk across different populations and subtypes. The studies consistently show that prolonged breastfeeding reduces the risk of TNBC and basal-like tumors, mitigates the parity-associated increase in ER-negative disease, and provides additional protection in BRCA1 carriers. Experimental models further support the notion that abrupt involution, mimicking a lack of breastfeeding, induces inflammatory and progenitor cell changes that may predispose individuals to TNBC. (Abbreviations: AA, African-American; AF, attributable fraction; BF, breastfeeding; PAF, population attributable fraction; WHR, waist-to-hip ratio; ER, estrogen receptor; PR, progesterone receptor; HER2, human epidermal growth factor receptor 2; BRCA, breast cancer susceptibility gene; CI, confidence interval; OR, odds ratio; HR, hazard ratio).

Study (Year) [Ref.]	Design/Population	Exposure Metric	Outcome/Subtype	Effect Size (95% CI)
Collaborative Group, Lancet (2002) [35]	Collaborative re-analysis, 47 studies (30 countries)	Per 12 months of breastfeeding (lifetime)	All breast cancer	RR↓ 4.3% (95% CI 2.9–5.8; *p* < 0.0001) per 12 months; ↓7% (5.0–9.0; *p* < 0.0001) per birth
Islami et al., Ann. Oncol. (2015) [28]	Systematic review & meta-analysis (27 studies; 36,881 cases)	Ever vs. never breastfeeding (parous women)	TNBC	OR 0.78 (0.66–0.91)
Ma, et al., Breast Cancer Res. (2017) [20]	Pooled analysis of 3 population-based case-control studies	≥1 year vs. never (parous)	TNBC	OR 0.69 (0.50–0.96)
John, et al., BEM Study, Int. J. Cancer (2018) [26]	Multiethnic case-control (<50 y focus)	≥24 mo vs. 0 mo (parous, <50 y)	TNBC	OR 0.52 (0.26–1.04) (borderline)
Kotsopoulos, et al., Cancer Res. (2012) [29]	Case-control, BRCA carriers	≥1 y vs. <1 y; ≥2 y	BRCA1-associated BC	OR 0.68 (0.52–0.91); ≥2 y: OR 0.51 (0.35–0.74)
Chehayeb, et al., NPJ Breast Cancer (2025) [6]	Population-level modelling (US)	Attributable fraction estimate	TNBC	AF: ~15% (Black) & ~12% (White) potentially avoidable with BF support
AMBER Consortium (Palmer, et al., 2014, JNCI) [24]	Multi-study consortium of African-American women	Parity × breastfeeding (ever/never; by number of births)	ER- and TNBC	Parous vs. nulliparous: OR 1.33 (1.11–1.59) for ER-; among never-breastfed, ≥4 y vs. 1 birth OR 1.68 (1.15–2.44); breastfeeding attenuated the parity-associated ER- risk
Black Women’s Health Study (Palmer, et al., 2011, CEBP) [22]	Prospective cohort (AA women)	Parity; breastfeeding (ever/never)	ER-/PR-	3+ vs. 0 births: HR 1.48 (0.98–1.84) for ER-/PR-; among women who had breastfed, high parity was no longer associated with increased ER-/PR- incidence
Carolina Breast Cancer Study (Millikan et al., 2008, BCRT) [27]	Population-based case-control	Never vs. ever breastfeeding; central adiposity (WHR)	Basal-like (≈TNBC)	Joint PAF ≈53% For Basal-Like from Never breastfeeding + elevated WHR; up to 68% preventable in younger AA women with breastfeeding promotion & adiposity reduction
Pregnancy-associated TNBC (ElShamy, 2016, Oncotarget) [34]	Narrative synthesis + experiment	Breastfeeding duration	Pregnancy-associated TNBC	Summarizes evidence that longer lactation lowers risk; cites does-response for overall BC (~4.3% lower RR per 12 months BF)

## Data Availability

No new data were created or analyzed in this study.

## Supplementary Materials

The following supporting information can be downloaded at: https://www.mdpi.com/article/10.3390/pathogens14090946/s1, Table S1. Evidence from Breast, Milk, and Gut Microbiota Studies in Relation to Breast Cancer.
