# Supplementary material for: Long-Term Breastfeeding: Protective Effects Against Triple-Negative Breast Cancer and the Role of the Breast Microbiota
Source: Pathogens. 2025 Sep 18;14(9):946. doi: 10.3390/pathogens14090946 (PMC12473010; doi:10.3390/pathogens14090946)
Supplement: Supplementary file 1 [file pathogens-14-00946-s001.zip › pathogens-3825423-supplementary.pdf]

Supplementary Table S1. Evidence from Breast, Milk, and Gut Microbiota Studies in Relation to Breast Cancer

This supplementary table compiles evidence from studies investigating the microbiota of breast tissue, human milk, and the gut–breast axis. For each domain, we summarize study population, methods, and key findings relevant to breast cancer and TNBC.

Part A summarizes studies of the breast tissue and tumor microbiota, highlighting microbial diversity, subtype-specific patterns, and associations with TNBC risk and progression.

Part B includes studies of the human milk microbiota, showing how maternal and infant factors, as well as feeding practices, shape microbial communities with potential implications for maternal breast health.

Part C covers the gut–breast axis, including estrobolome function, SCFA production, and diet- or probiotic-based interventions, emphasizing mechanisms by which the gut microbiota may influence breast cancer risk and therapy response.

Part A. Breast Tissue / Tumor Microbiota

Study (Author, Year)	Microbiome Source	Cohort/Sample	Methodology	Key Findings
**Urbaniak et al., 2014 (AEM)**	Breast tissue (tumor vs healthy)	n≈60 women	16S rRNA sequencing; culture-independent	Breast tissue harbors microbiota; tumors enriched in Proteobacteria/Firmicutes (Staphylococcus, Bacillus).
**Hieken et al., 2016 (Sci Rep)**	Breast tissue (benign vs malignant)	Surgically collected	16S rRNA sequencing; aseptic collection	Cancer tissues show reduced alpha diversity and distinct composition vs benign controls.
**Banerjee et al., 2018 (Front Microbiol)**	Breast tumors by subtype	Subtype-stratified tumors	16S rRNA sequencing	Subtype-specific microbial signatures; patterns differ between TNBC and luminal/HER2+ tumors.
**Smith et al., 2019 (Sci Rep)**	Breast tissue (race, stage, subtype)	NH-Black & NH-White women	16S rRNA sequencing	Microbiota differs by race, stage, and subtype; subtype- and disparity-linked shifts.

**Hoskinson et al., 2022 (mSystems)**	Mammary microbiota (pre/post diagnosis)	Longitudinal sampling	16S / shotgun functional profiling	Functional potential shifts with tumorigenesis trajectory; suggests early microbial changes.
**German et al., 2023 (BCR)**	Breast tissue cohort	Large cohort profiling	16S and/or shotgun	Breast microbial composition associates with classic BC risk factors.
**Gerbec et al., 2025 (mBio)**	Intratumoral bacteria (TNBC)	Patient-derived isolates & models	Isolation, genomics, functional assays	Bacillus thermoamylovorans enhances TNBC metastasis; higher Bacillus linked to worse survival.
**Rad et al., 2025 (Microorganisms)**	Breast tissue (meta-analysis)	Multiple datasets (normal/mastitis/cancer)	Systematic review & meta-analysis	↓ Alpha diversity in tumors; enrichment of Fusobacteriota, Peptoniphilus, Atopobium; strongest in TNBC pooled analyses.
**Urbaniak et al., 2016 (AEM)**	Breast tissue follow-up	Independent set	16S rRNA sequencing	Further supports the presence of a tissue microbiome and differential abundance in cancer.

Part B. Human Milk Microbiota

Study (Author, Year)	Microbiome Source	Cohort/Sample	Methodology	Key Findings
**Moossavi et al., 2019 (Cell Host Microbe)**	Human milk	393 mother–infant dyads (CHILD cohort)	16S rRNA sequencing; causal modeling	Feeding mode is a major driver: direct breastfeeding enriches Lactobacillus/Bifidobacterium/Veillonella; pumped milk enriches opportunists.
**Pannaraj et al., 2017 (JAMA Pediatr)**	Milk & infant stool	Mother–infant pairs; longitudinal	16S rRNA; source tracking	Milk bacteria seed infant gut microbiota; supports entero-mammary/retrograde transfer routes.
**Williams et al., 2017 (J Hum Lact)**	Human milk (multi-omics)	Cross-sectional	Microbiota + HMOs + macronutrients	Milk communities correlate with oligosaccharides and maternal cells; host–nutrient context shapes ecosystem.
**Togo et al., 2019 (Future Microbiol)**	Breast & milk microbiota	Systematic review	Synthesis of 16S studies	Catalogs taxa and variability; underscores contamination

				control challenges in low-biomass samples.
**McGuire & McGuire, 2015 (Adv Nutr)**	Human milk	Narrative review	Conceptual synthesis	Positions human milk as a probiotic ecosystem delivering beneficial microbes.
**Rodríguez, 2014 (Adv Nutr)**	Entero-mammary pathway	Narrative review	Mechanistic overview	Proposes maternal gut-to-mammary translocation; provides biological plausibility for BF–microbiota link.

Part C. Gut–Breast Axis and Therapy

Study (Author, Year)	Microbiome Source	Cohort/Sample	Methodology	Key Findings
**Fuhrman et al., 2014 (JCEM)**	Fecal microbiome & estrogen metabolism	Postmenopausal women	16S rRNA; urinary estrogen metabolites	Gut microbiota (estrobolome) associates with estrogen metabolite profiles; pathway for hormone-related risk modulation.
**Shively et al., 2018 (Cell Reports)**	Mammary microbiome (diet manipulation)	Non-human primate model	Dietary intervention (Mediterranean vs Western)	Dietary pattern modulates mammary microbiome; demonstrates modifiability of breast ecosystem.
**Laborda-Illanes et al., 2020 (Cancers)**	Gut & breast microbiota mechanisms	Comprehensive review	Mechanistic synthesis	Links SCFAs, β-glucuronidase, immunity, and therapy response across gut–breast axis; relevance to TNBC.
**Nat Med, 2014 (Chemo–Immunity–Microbiota)**	Microbiota ↔ therapy immunity	Perspective/commentary	Conceptual analysis	Microbiota modulates host responses to anticancer therapy; implications for immunotherapy in BC/TNBC.
**Pellegrini et al., 2020 (Nutrition)**	Gut microbiota intervention	Overweight BC survivors; pilot RCT	Diet + probiotics	Feasibility of microbiome-targeted interventions; exploratory improvements in metabolic/inflammatory markers.
**Pathogens, 2023 (β-glucuronidase review)**	Gut microbial enzyme target	Review	Mechanistic synthesis	β-Glucuronidase as a potential therapeutic target to modulate estrogen reactivation and BC risk.

**IJO 2024; STTT 2023 (metabolites & therapy)**	Microbial metabolites & cancer therapy	Reviews	Translational synthesis	Microbial metabolites reshape tumor microenvironment and influence therapy outcomes; implications for TNBC immunotherapy.

References for Supplementary Table S1

1. Urbaniak et al., Appl Environ Microbiol 2014.

2. Hieken et al., Sci Rep 2016.

3. Banerjee et al., Front Microbiol 2018.

4. Smith et al., Sci Rep 2019.

5. Hoskinson et al., mSystems 2022.

6. German et al., Breast Cancer Res 2023.

7. Gerbec et al., mBio 2025.

8. Rad et al., Microorganisms 2025.

9. Urbaniak et al., Appl Environ Microbiol 2016.

10. Moossavi et al., Cell Host Microbe 2019.

11. Pannaraj et al., JAMA Pediatr 2017.

12. Williams et al., J Hum Lact 2017.

13. Togo et al., Future Microbiol 2019.

14. McGuire & McGuire, Adv Nutr 2015.

15. Rodríguez, Adv Nutr 2014.

16. Fuhrman et al., J Clin Endocrinol Metab 2014.

17. Shively et al., Cell Reports 2018.

18. Laborda-Illanes et al., Cancers 2020.

19. Karin et al., Nat Med 2014.

20. Pellegrini et al., Nutrition 2020.

21. Pathogens 2023 (β-glucuronidase review).

22. Int J Oncol 2024; Signal Transduct Target Ther 2023.
